# Simple Method for Shiga Toxin 2e Purification by Affinity Chromatography via Binding to the Divinyl Sulfone Group

**DOI:** 10.1371/journal.pone.0083577

**Published:** 2013-12-10

**Authors:** Hideyuki Arimitsu, Keiko Sasaki, Hiroe Kojima, Tadashi Yanaka, Takao Tsuji

**Affiliations:** 1 Department of Microbiology, Fujita Health University School of Medicine, Toyoake, Aichi, Japan; 2 Advanced Technology Development Center, Kyoritsu Seiyaku Corporation, Tsukuba, Ibaraki, Japan; CNR, Italy

## Abstract

Here we describe a simple affinity purification method for Shiga toxin 2e (Stx2e), a major causative factor of edema disease in swine. *Escherichia coli* strain MV1184 transformed with the expression plasmid pBSK-Stx2e produced Stx2e when cultivated in CAYE broth containing lincomycin. Stx2e bound to commercial D-galactose gel, containing α-D-galactose immobilized on agarose resin via a divinyl sulfone linker, and was eluted with phosphate-buffered saline containing 4.5 M MgCl_2_. A small amount of Stx2e bound to another commercial α-galactose-immobilized agarose resin, but not to β-galactose-immobilized resin. In addition, Stx2e bound to thiophilic adsorbent resin containing β-mercaptoethanol immobilized on agarose resin via a divinyl sulfone, and was purified in the same manner as from D-galactose gel, but the Stx2e sample contained some contamination. These results indicate that Stx2e bound to D-galactose gel mainly through the divinyl sulfone group on the resin and to a lesser extent through α-D-galactose. With these methods, the yields of Stx2e and attenuated mutant Stx2e (mStx2e) from 1 L of culture were approximately 36 mg and 27.7 mg, respectively, and the binding capacity of the D-galactose gel and thiophilic adsorbent resin for Stx2e was at least 20 mg per 1 ml of resin. In addition, using chimeric toxins with prototype Stx2 which did not bind to thiophilic adsorbent resin and some types of mutant Stx2e and Stx2 which contained inserted mutations in the B subunits, we found that, at the least, asparagine (amino acid 17 of the B subunits) was associated with Stx2e binding to the divinyl sulfone group. The mStx2e that was isolated exhibited vaccine effects in ICR mice, indicating that these methods are beneficial for large-scale preparation of Stx2e toxoid, which protects swine from edema disease.

## Introduction

Shiga toxin-producing *Escherichia coli* (STEC) strains produce Shiga toxin (Stx), which is associated with hemolytic uremic syndrome (HUS) in humans [1]. Stx is mainly classified into Stx1 and Stx2. Each toxin consists of an enzymatically active A subunit and a pentameric association of B subunits responsible for the binding to glycolipid receptors. In addition to prototype Stx2, there are four types of variants of Stx2 based on differences in amino acid sequences, namely Stx2c, Stx2d, Stx2e, and Stx2f [2-6]. Stx2e is a major causative factor of edema disease, which is mainly observed in piglets 1 to 2 weeks after weaning. Typical clinical signs of edema disease are well documented [7-9] and include edema of the eyelids and neurological disorders such as ataxia, convulsions, and paralysis. Although the occurrence of edema disease is sporadic and morbidity is low (approximately 16%), mortality is considerably higher (approximately 64%). In addition, since the recurrence of the disease occurs in some herds due to persistence of the bacteria, the economic damage caused by this disease is serious for swine farmers.

The identity of the amino acid sequences of the A and B subunits shared between Stx2 and Stx2e are approximately 94% and 84%, respectively, and the binding properties of the B subunits to glycolipids also differ; Stx2 binds only to globotriaosylceramide (Gb3; Galα1-4Galβ1-4GlcCer), whereas Stx2e binds preferentially to globotetraosylceramide (Gb4; GalNAcβ1-3Galα1-4Galβ1-4GlcCer) and weakly to Gb3 [10,11]. 

Several reports have demonstrated that attenuated Stx2e toxoids are good vaccine antigens that can protect piglets from edema disease [12-15]. However, conventional methods for purifying Stx2e are considerably cumbersome, requiring a large volume of culture and several chromatography steps [16-18]. Therefore, the development of effective purification methods for Stx2e would be beneficial for large-scale preparation of Stx2e vaccine antigen. Gb3-immobilized resin and avian ovomucoid glycoprotein/glycopeptides-immobilized resin, which are used to purify Shiga toxins, are applicable to the purification of Stx1 [19-21]. Moreover, Stx2e can easily be purified from P1 glycoprotein isolated from hydatid cyst material, which contains the Galα1-4Gal structure [22]. However, since the purification yield from this process is not high enough (0.16 mg from 1 L of culture), there is a need to utilize overexpression and develop an improved, simple purification method for Stx2e that is cost effective.

In this study, we attempted to purify Stx2e by using various commercial resins, each of which contained an immobilized sugar component of Gb4, and found that Stx2e specifically bound to α-D-galactose-immobilized resin. In the process of developing this purification method, we found that Stx2e bound mainly to the divinyl sulfone group rather than to α-galactose on agarose resin. In addition, we identified the amino acid residue of Stx2e that was associated with the interaction with the divinyl sulfone group.

## Materials and Methods

### Construction of expression plasmid　

**Figure 1:**
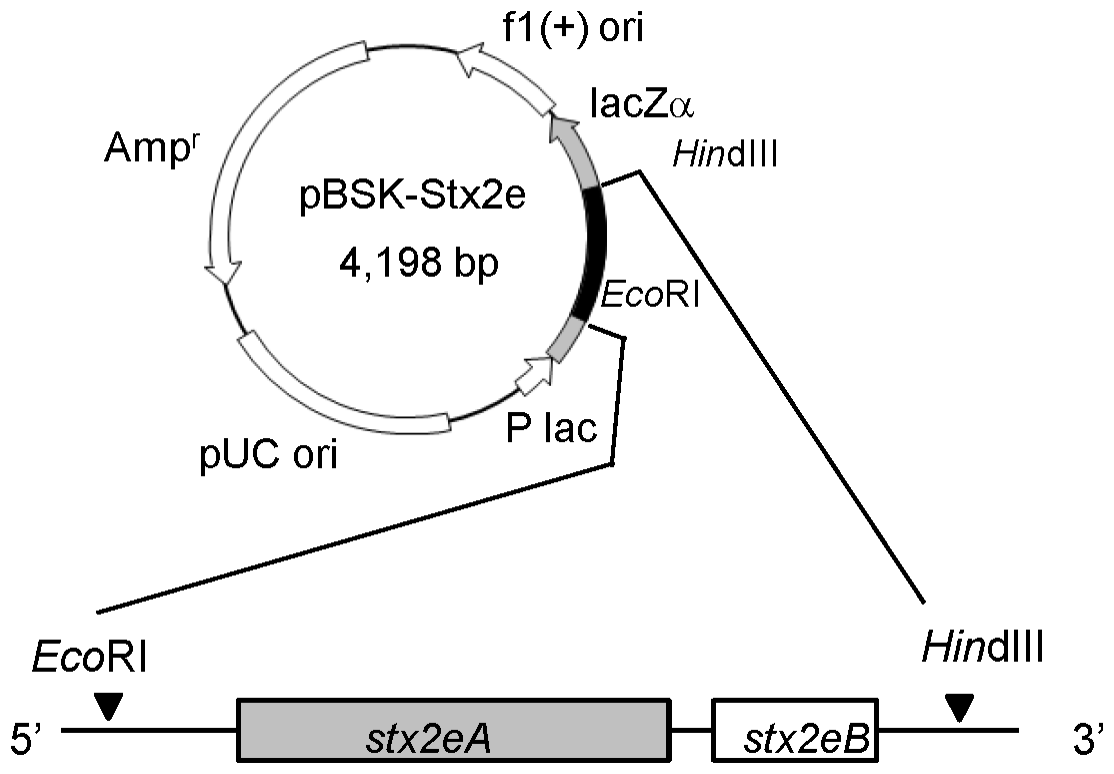
Map of expression plasmid of Stx2e. A full-length *Stx2e* gene was inserted into the *lacZ*α gene fragment of pBluescript II SK(+) in a different reading frame using the restriction sites *Eco*RI and *Hin*dIII.

The expression plasmid was constructed according to previously described methods [23,24]. STEC strain 220811A78 (serogroup O139, F18+), which was isolated from piglets with edema disease, was kindly provided by Kyoritsu Seiyaku Corporation (Tokyo, Japan). The *Stx2e* gene was polymerase chain reaction (PCR)-amplified using genomic DNA from the 220811A78 strain as a template and primer sets LTB(SD)Stx2e(EcoRI)-f and Stx2eB(HindIII)-r. The amplified product was cloned into the pMD20-T vector (Promega, Madison, WI) and then transformed into *E. coli* strain TOP10F’. After confirming the sequence with that of *Stx2e* registered in the database (accession number GU459254), the cloned DNA extracted from the plasmid using the restriction enzymes *Eco*RI and *Hin*dIII was subcloned into the pBluescript II SK(+) vector (Stratagene, La Jolla, CA) digested with the same enzymes. The resultant plasmid (shown in Figure 1) was designated pBSK-Stx2e. The expression plasmid of prototype Stx2 (pBSK-Stx2) was generated from pBSK-Stx2(His), which was an expression plasmid of histidine-tagged Stx2 [24], by site-directed mutagenesis using a QuikChange II Site-directed Mutagenesis Kit (Stratagene) and the primer set Stx2(his-)-f and Stx2(his-)-r to separate the histidine-tag gene from 3’ end of the B subunit gene. The expression plasmids of the mutant toxins were generated from either pBSK-Stx2e or pBSK-Stx2 by site-directed mutagenesis. The expression plasmids of the chimeric toxins (Stx2eA2B and Stx2A2eB) were generated using a GENEART Seamless Cloning and Assembly Kit (Invitrogen, Life Technologies Corp, Carlsbad, CA). All primer sequences used in this study are listed in Table S1.

### Protein Expression

Protein expression was conducted according to previously described methods [23,24]. Briefly, *E. coli* strain MV1184 was transformed with each plasmid and then cultured in CAYE broth containing 50 μg/ml of ampicillin and 90 μg/ml of lincomycin (Pfizer, New York, NY) for 48 h at 30°C. After centrifugation, the harvested cells were suspended and sonicated in phosphate-buffered saline (PBS) containing 0.5 M NaCl. The cell extract was collected from sonicated cells by centrifugation (15,000 × g, 100 min) and was used to investigate the binding ability of Shiga toxins to sugar-immobilized resin and thiophilic adsorbent resin (TAR), or to purify Stx2e, as described below.

### Evaluation of toxin binding to resins

To investigate the binding ability of Stx2e or Stx2 to sugar-immobilized resin or TAR, 25 μl of D-galactose gel (Immobilized D-Galactose Gel; Thermo Fisher Scientific, Rockford, IL), TAR (Thiophilic Adsorbent; Thermo Fisher Scientific), lactose gel (Lactose Gel; EY Laboratories, San Mateo, CA), α-galactose gel (α-Galactose Gel; EY Laboratories), β-galactose gel (β-Galactose Gel; EY Laboratories), or N-acetylgalactosamine gel (N-Acetylgalactosamine Gel; EY Laboratories) was washed with 1 ml of PBS containing 0.5 M NaCl in a 1.5 ml microtube, followed by the addition of 50 μl of cell extract. After washing three times with 1 ml of the same buffer, the resin was mixed with 25 μl of 2 × SDS-PAGE sample buffer and heated in boiling water for 5 min. A total of 10 μl of each sample was separated by SDS-PAGE, and the gel was stained with Coomassie brilliant blue (CBB)-R250 or analyzed by western blot analysis.

### Large-scale purification of Stx2e

For purification of Stx2e, cell extract from 1 L of culture was applied onto a 2-ml volume of D-galactose gel or TAR column equilibrated with PBS containing 0.5 M NaCl. After washing the column, bound protein was eluted with PBS containing 4.5 M MgCl_2_, and 2 ml of each fraction was collected. The eluent was pooled and dialyzed against PBS overnight, and the precipitated products were removed by centrifugation.

Protein concentrations were determined with DC protein assay reagent (Bio-Rad, Hercules, CA) using bovine serum albumin as a standard.

### Preparation of rabbit antiserum

To generate antiserum for the detection of Stx2e and Stx2 in bacterial cell extracts, female Japanese White rabbits (13 weeks old, Japan SLC, Hamamatsu, Japan) were immunized with either genetically attenuated mutant Stx2e (mStx2e) or histidine-tagged mutant Stx2 (mStx2-His) [24], each with a glutamic acid at 167 and arginine at 170 in the A subunit which was replaced by glutamine and leucine (E167Q+R170L), respectively. Initially, 400 μg of each protein was emulsified with Freund’s complete adjuvant and injected subcutaneously. Two weeks later, each rabbit was given a second subcutaneous immunization of the same dose of mutant toxin with Freund’s incomplete adjuvant. After 2 additional weeks, a final booster injection of 100 μg of mutant toxin was administered intravenously without adjuvant. One week later, a whole blood sample was collected from the carotid artery under anesthesia with pentobarbital sodium, and the antiserum was collected by centrifugation at 3,000 × g for 20 min. 

### Toxicity assays

For *in vitro* assays, Vero cells were plated into each well of a 96-well culture plate at 1 × 10^4^ /0.1 ml of minimum essential medium containing 10% fetal calf serum with penicillin and streptomycin and incubated overnight at 37°C. After removing the supernatant, diluted toxins were added, and the cells were incubated for 72 h at 37°C. Cytotoxicity was measured according to the method of Gentry et al., which was evaluated using the crystal violet staining method [25]. For *in vivo* assays, wild-type and mutant Stx2e were serially diluted with PBS, and 0.5 ml of each dilution was injected intraperitoneally into at least 5 female ICR mice (6 weeks old, Japan SLC). The animals were observed for 1 week and deaths were recorded. The median lethal dose (LD_50_) was calculated from the dilution that caused the death of half of the animals.

### Vaccination of mice with mStx2e

Evaluation of the vaccine effect of mStx2e (E167Q+R170L) in mice was conducted according to that of mStx2-His reported previously [24]. Briefly, 1 μg of mStx2e containing 0.05% (w/v) aluminum hydroxide as an adjuvant in 0.2 ml of PBS was subcutaneously injected into 15 female ICR mice (6 weeks old) twice, with a 3-week interval in between vaccinations. For a control group, PBS containing 0.05% (w/v) aluminum hydroxide instead of mStx2e was injected into 5 mice. Two weeks after the secondary immunization, the animals were tail bled to determine the specific antibody titer in the sera by enzyme-linked immunosorbent assay (ELISA). The mice immunized with mStx2e were divided into three groups (n=5 per group), and all mice were intraperitoneally challenged with 1, 10, or 100 μg of Stx2e (corresponding to 20, 200, or 2,000-fold LD_50_, respectively) in 0.5 ml of PBS, respectively, and their survivability was monitored for 1 week. All animal experiments were carried out in strict accordance with the recommendations in the Regulations for the Management of Laboratory Animals at Fujita Health University. All procedures were approved by the Institutional Animal Care and Use Committee of Fujita Health University (Permit Number: M2371). All efforts were made to minimize suffering.

### Determination of Stx2e-specific IgG titers

Stx2e-specific antibody titers in the mice sera were determined by ELISA according to the Stx2-His antibody titer method described previously [24], and the data were statistically analyzed using a Student’s t-test between the mStx2e and adjuvant groups.

### Sodium dodecyl sulfate-polyacrylamide gel electrophoresis (SDS-PAGE) and western blot analysis

The sample proteins were resolved on a 15% polyacrylamide gel. The gel was stained with CBB-R250 or electroblotted onto a polyvinylidene difluoride (PVDF) membrane using the iBlot gel transfer system (Invitrogen). Western blot analysis was conducted for detecting wild-type or mutant toxins (Stx2e and Stx2) in the bacterial cell extracts with rabbit antiserum. After washing with PBS containing 0.05% Tween 20 (T-PBS), each membrane was incubated with T-PBS containing 5% skim milk (S-PBS) for 1 h and then reacted with either anti-mStx2e rabbit serum or anti-mStx2-His rabbit serum diluted with T-PBS containing 5% bovine serum albumin for 1 h. Then, each membrane was incubated for 1 h with horseradish peroxidase (HRP)-conjugated anti-rabbit immunoglobulins porcine immunoglobulin (Dako, Copenhagen, Denmark) diluted with S-PBS. Specific bands were detected with the Immobilon Western Chemiluminescent HRP Substrate (Millipore, Billerica, MA) using an LAS4000 image analyzer (Fujifilm, Tokyo, Japan). All reactions were carried out at room temperature, and the membrane was washed three times with T-PBS for 5 min before each reaction. SDS-PAGE Molecular Weight Standards (Broad Range, Bio-Rad) and MagicMark XP Western Protein Standard (Invitrogen) were used for molecular weight size markers for SDS-PAGE and western blot analysis, respectively.

The N-terminal amino acid sequence of each subunit on the PVDF membrane stained with CBB-R250 was determined with a pulsed-liquid phase protein sequencer (Procise 491HT; Applied Biosystems, Life Technologies Corp, Carlsbad, CA).

## Results

### Stx2e specifically binds to D-galactose gel

**Figure 2:**
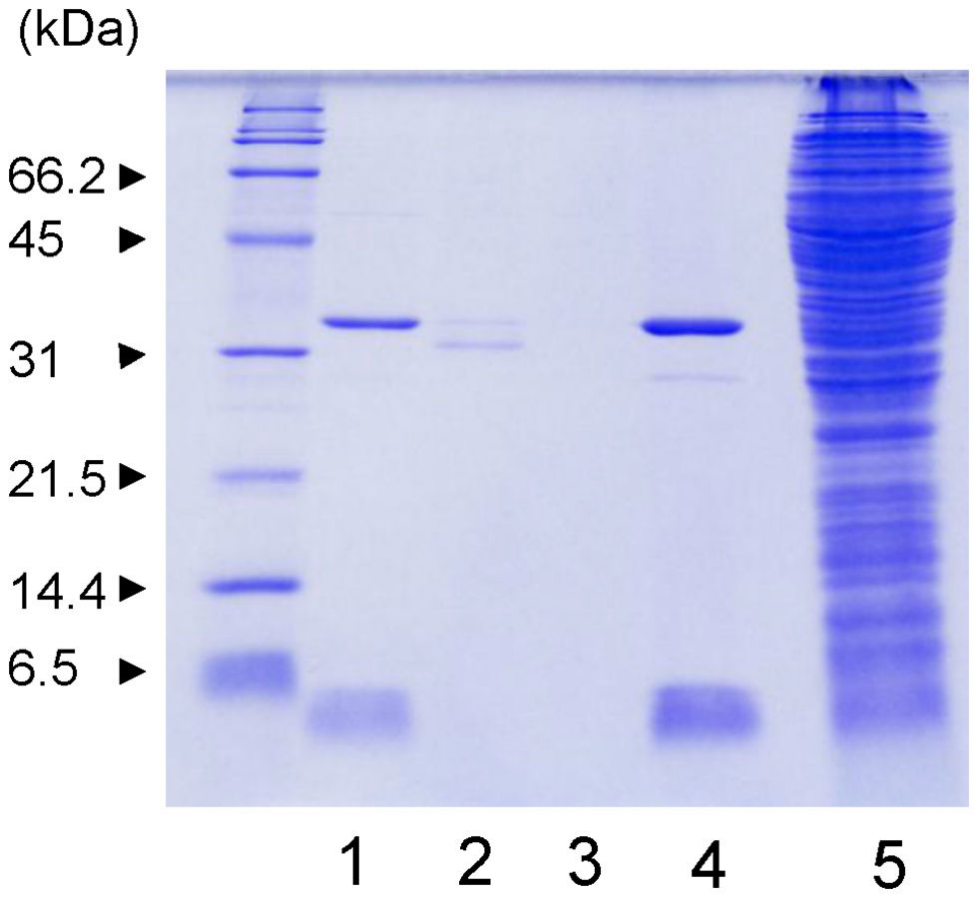
Binding of Stx2e to sugar-immobilized resin. Fifty microliters of cell extract containing Stx2e was added to each 25 μl of D-galactose gel (lane 1), lactose gel (lane 2), and N-acetylgalactosamine gel (lane 3). After washing the resins with PBS containing 0.5 M NaCl, 25 μl of 2 × SDS-PAGE sample buffer was added to each gel pellet, and the samples were heated for 5 min. Each 10 μl sample was loaded onto the 15% (w/v) polyacrylamide gel. Five micrograms of purified Stx2e and 5 μl of the cell extract before addition to the resins were added to lanes 4 and 5, respectively, and 5 μl of molecular weight size marker was loaded in the lane on the left side.

Firstly, we prepared an expression plasmid for Stx2e (pBSK-Stx2e) and then searched for an affinity resin to effectively purify Stx2e protein. Based on the binding profile of Stx2e to Gb4 (GalNAcβ1-3Galα1-4Galβ1-4GlcCer), we focused on D-galactose, lactose (Galβ1-4Glc), and N-acetylgalactosamine (GalNAc) as ligands for affinity purification and investigated whether Stx2e bound to the small volume (25 μl) of agarose resin containing each sugar immobilized to the resin. After the resin was reacted with 50 μl of cell extract and washed, the bound protein was dissociated from the resin by heating with SDS-PAGE sample buffer and then analyzed by SDS-PAGE. As shown in Figure 2, the D-galactose gel specifically bound Stx2e but other sugar-immobilized resin did not.

### Large -Scale Purification of Stx2e

**Figure 3:**
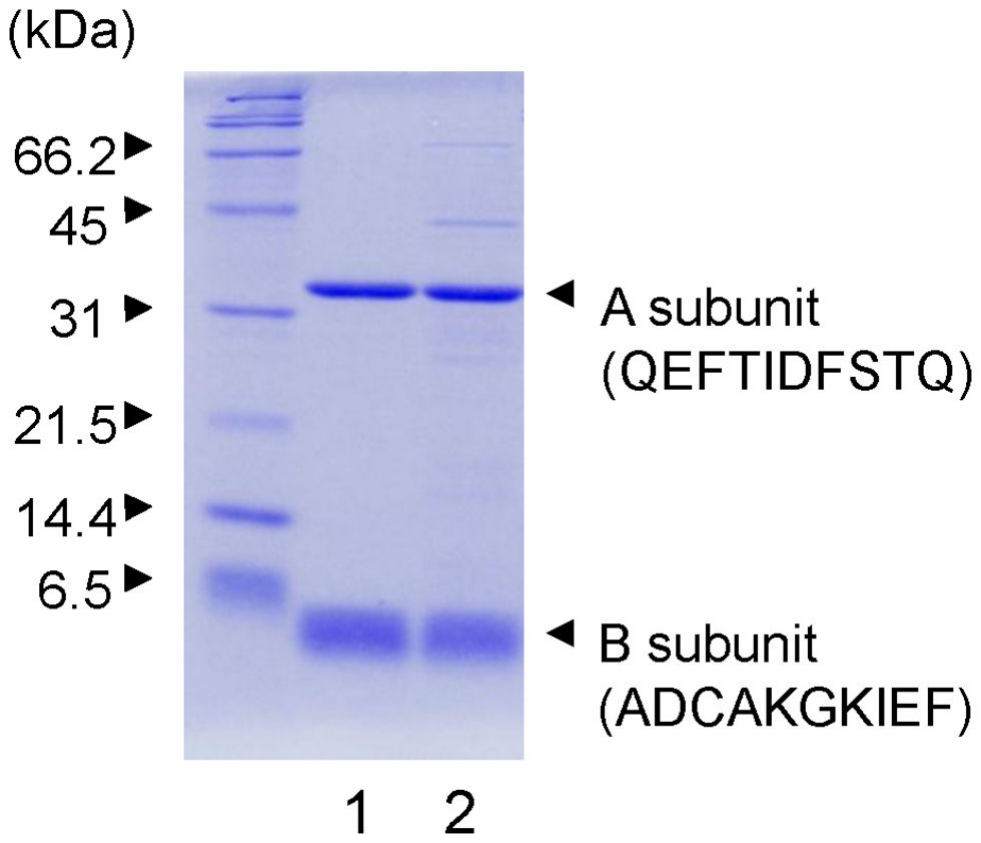
SDS-PAGE profile of Stx2e purified with D-galactose gel resin (lane 1) and thiophilic adsorbent resin (lane 2). Five micrograms of each purified toxin was loaded onto the 15% polyacrylamide gel and stained with Coomassie brilliant blue R-250. The molecular weight size marker was loaded in the lane on the left side. The sequence of the ten N-terminal amino acids of each band is shown in parentheses.

To determine the optimal conditions for purifying Stx2e using D-galactose gel, we applied the cell extract containing Stx2e onto a 2-ml D-galactose gel column and then applied various buffers to the column containing bound Stx2e. Among the buffers investigated, only PBS containing 4.5 M MgCl_2_ dissociated Stx2e from the D-galactose gel, whereas PBS containing 0.3 M D-galactose or lactose or 0.1 M glycine-HCl (pH 3.0) did not. Based on the elution profile, we purified Stx2e from 1 L of culture in CAYE broth. After eliminating a faint precipitate that formed during the dialysis step against PBS, Stx2e was completely purified, as only A and B subunit bands were visible in lane 1 of the gel shown in Figure 3. The N-terminal amino acid sequence of each band was identical to the deduced sequence reported by Gyles et al. and Weinstein et al [5,6]. The purification yield of Stx2e was approximately 36 mg from 1 L of culture in CAYE broth, and the binding capacity of the D-galactose gel for Stx2e was at least 20 mg from 1 ml of resin. This method was also used to purify attenuated mStx2e (E167Q+R170L); the purification yield was approximately 27.7 mg.

### Stx2e binds mainly to the divinyl sulfone group and somewhat to α-galactose

**Figure 4:**
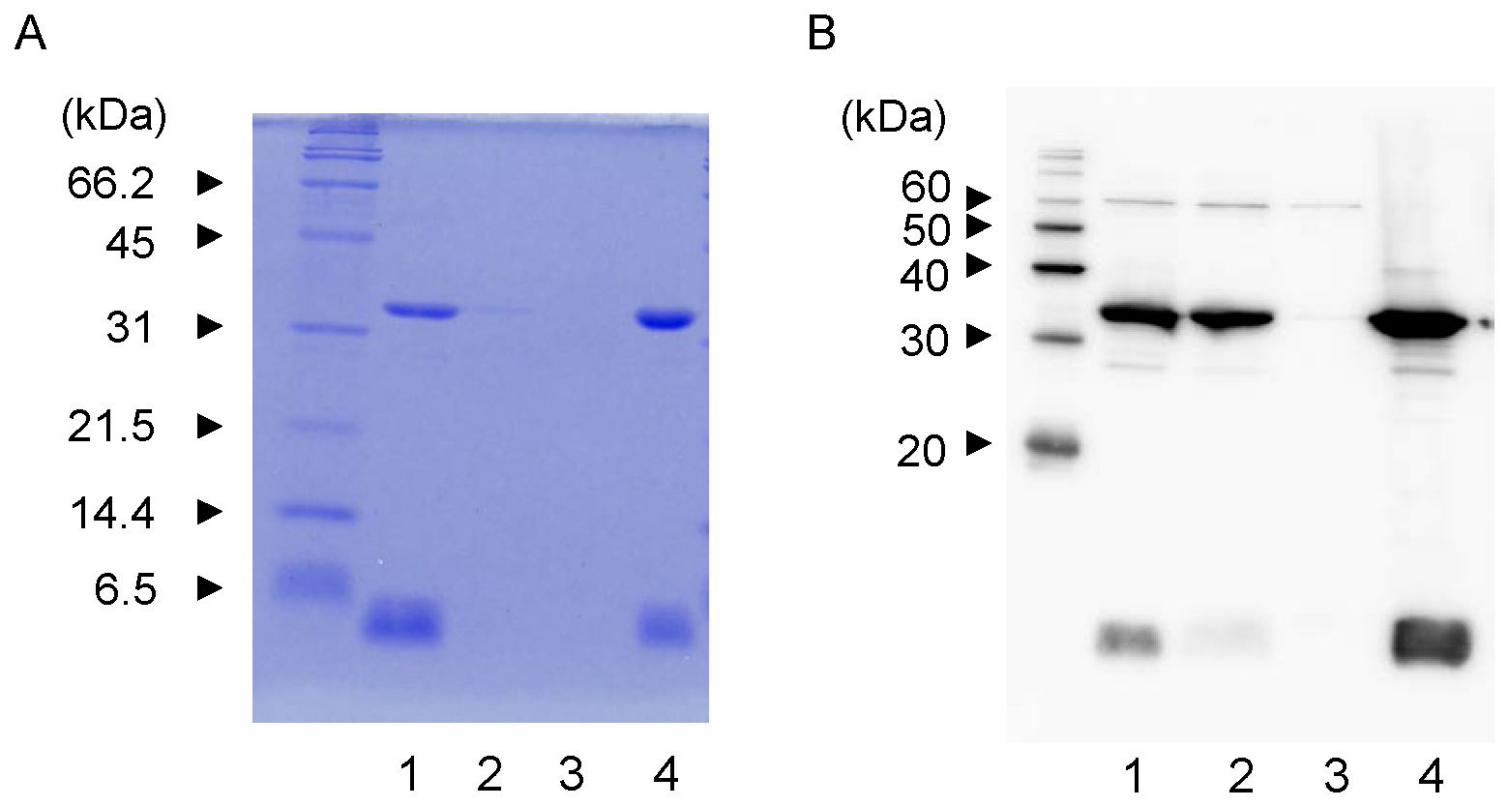
Binding of Stx2e to other galactose-immobilized resins. Fifty microliters of the cell extract containing Stx2e was added to each 25 μl of D-galactose gel (from Thermo Fisher Scientific, lane 1), α-galactose gel (from EY Laboratories, lane 2) and β-galactose gel (from EY Laboratories, lane 3). After washing the resins with PBS containing 0.5 M NaCl, 25 μl of 2 × SDS-PAGE sample buffer was added to each gel pellet, and the samples were heated for 5 min. Each 10 μl sample was loaded onto the 15% (w/v) polyacrylamide gel. The gel was stained with CBB-R250 (A) or electroblotted onto PVDF membrane and subjected to western blot analysis using anti-Stx2e rabbit serum (B). Five micrograms of purified Stx2e (positive control) was loaded in lane 4, and 5 μl of molecular weight size marker was loaded in the lane on the left side.

To investigate whether Stx2e bound to other galactose-immobilized resins, other commercial resins (from EY Laboratories) were employed for Stx2e binding. As shown in Figure 4, we found that α-galactose gel bound Stx2e, but the binding level of Stx2e for α-galactose gel was lower than that for D-galactose gel. On the other hand, β-galactose gel did not bind Stx2e, even at the detection level of western blot analysis (Figure 4B). The coupling ligand of D-galactose gel is also α-galactose, indicating that Stx2e recognizes not only α-galactose, but also other structures present in the D-galactose gel.

The affinity purification method of immunoglobulins by TAR was first reported by Porath et al. [26]. This resin has a divinyl sulfone group, which is a reagent for coupling various ligands for affinity chromatography, as well as β-mercaptoethanol (2ME), which is originally used for blocking the residual divinyl sulfone group [26]. According to the manufacturer, D-galactose gel has a similar structure to that of TAR except for the tethered α-galactose or 2ME leading to the speculation that Stx2e bound to the divinyl sulfone group of these resins. Therefore, we investigated whether Stx2e could be purified by TAR under the same buffer conditions. As shown in lane 2 of Figure 3, Stx2e in the cell extract bound to TAR to the same degree as it bound to D-galactose gel (lane 1), but some minor contamination was present in the TAR-purified sample. The purification yield of Stx2e (including minor contaminants) using TAR was almost the same (approximately 36.5 mg from 1 L of culture in CAYE broth) as that obtained from purification in D-galactose gel.

These results indicate that Stx2e binds mainly to the divinyl sulfone group on resin and to a lesser extent to α-galactose, which suggests that α-galactose improves the specificity of D-galactose gel for Stx2e purification.

### Biological activity of Stx2e

To confirm the biological activity of purified Stx2e, we assayed *in vitro* and *in vivo* toxicity levels. The 50% cytotoxic dose (CD_50_) of Stx2e against Vero cells was approximately 6.4 pg/ml (1.69–14.29 pg/ml), whereas that of mStx2e was 6.9 μg/ml (3.29–10.09 μg/ml). In the *in vivo* assay, the LD_50_ of Stx2e against mice was 50 ng, whereas that of mStx2e was not determined, since none of the 6 mice examined died even after intraperitoneal injection of 100 μg of mStx2e.

### Stx2e binds to divinyl sulfone through its B subunits

**Figure 5:**
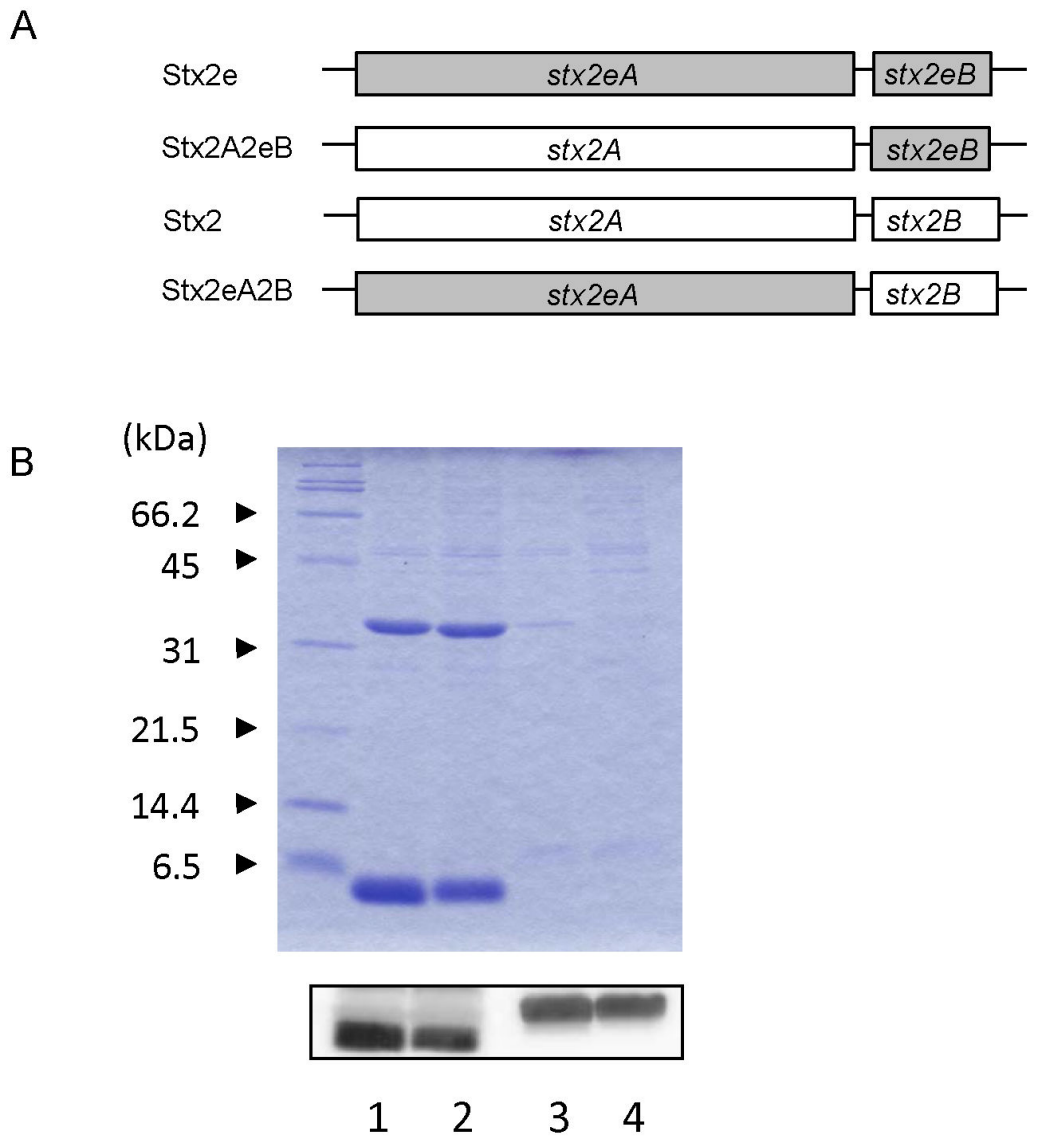
Binding of chimeric toxins of Stx2e and Stx2 to TAR. A) Diagram of genes encoding chimeric toxins. B) Binding of Stx2e, Stx2 and chimeric toxins to TAR (upper panel). A total of 10 μl of each TAR-binding product was loaded onto the 15% (w/v) polyacrylamide gel. Lanes 1; Stx2e, 2; Stx2A2eB, 3; Stx2, 4; Stx2eA2B. Expression of the B subunits in the cell extract of each transformant was detected by western blot analysis using anti-Stx2e (lane 1 and 2) or anti-Stx2 (lane 3 and 4) rabbit serum (lower panel).

We investigated whether TAR could be applied for the purification of prototype Stx2, which is a causative factor of HUS in human. However, Stx2 did not bind to TAR (Figure 5B, lane 3), suggesting that Stx2e bound to TAR through the amino acid residue that was intrinsic to Stx2e. Therefore, we prepared two types of expression plasmids that produced chimeric toxins: Stx2A2eB which consisted of the A subunit of Stx2 and the B subunits of Stx2e, and Stx2eA2B which consisted of the A subunit of Stx2e and the B subunits of Stx2 (Figure 5A). Stx2A2eB bound to TAR at the same level as Stx2e, but Stx2eA2B did not (Figure 5B), indicating that Stx2e binds to divinyl sulfone through its B subunits.

### Asparagine 17 is associated with the binding of Stx2e to divinyl sulfone

**Figure 6:**
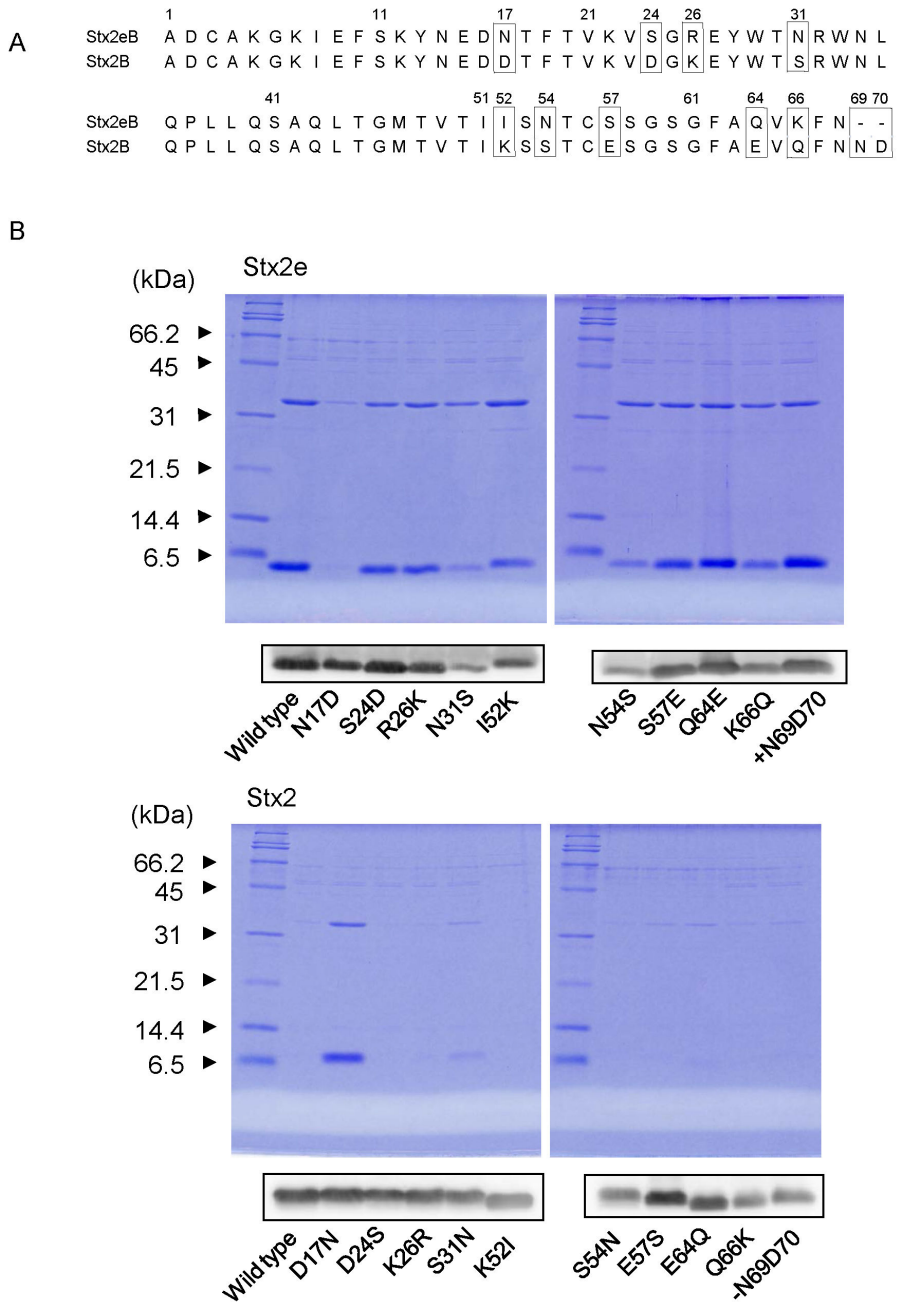
Identification of the amino acids in the B subunits associated with binding to divinyl sulfone. A) Comparative alignments of the amino acid sequences of mature Stx2eB and Stx2B. The amino acids that differ between the sequences are shown in boxes. B) TAR binding of each mutant Stx2e and Stx2 containing B subunits with introduced mutations. Each binding product was analyzed by SDS-PAGE and stained with CBB-R250 (upper panel). Expression of the B subunits in the cell extract of each transformant was detected by western blot analysis using anti-Stx2e or anti-Stx2 rabbit serum (lower panel).

To identify the amino acid residue of the B subunit of Stx2e that is associated with binding to TAR, we prepared mutant toxins whose B subunits had mutations and then investigated the binding of these toxins to TAR. As shown in Figure 6A, there are 9 amino acid differences between Stx2eB and Stx2B, and 2 amino acids (N69 and D70) are lacking in the C-terminal end of Stx2eB [5,6]. Therefore, we prepared 10 types of mutant Stx2e (N17D, S24D, R26K, N31S, I52K, N54S, S57E, Q64E, K66Q, and the C-terminal addition of 2 amino acids (+N69D70)) in which each different amino acid of Stx2eB was changed to that of Stx2B. Among these 10 types of mutant Stx2e, the binding of N17D to TAR was significantly reduced (Figure 6B). Furthermore, we prepared 10 types of mutant Stx2 (D17N, D24S, K26R, S31N, K52I, S54N, E57S, E64Q, Q66K, and the C-terminal deletion of 2 amino acids (-N69D70)) in which each different amino acid of Stx2B was changed to that of Stx2eB. In correspondence to the results of mutant Stx2e, only the D17N mutant of Stx2 significantly bound to TAR (Figure 6B), suggesting that, at the least, asparagine 17 in the B subunit was associated with the binding of Stx2e to divinyl sulfone.

### Vaccine effect of mStx2e in mice

**Figure 7:**
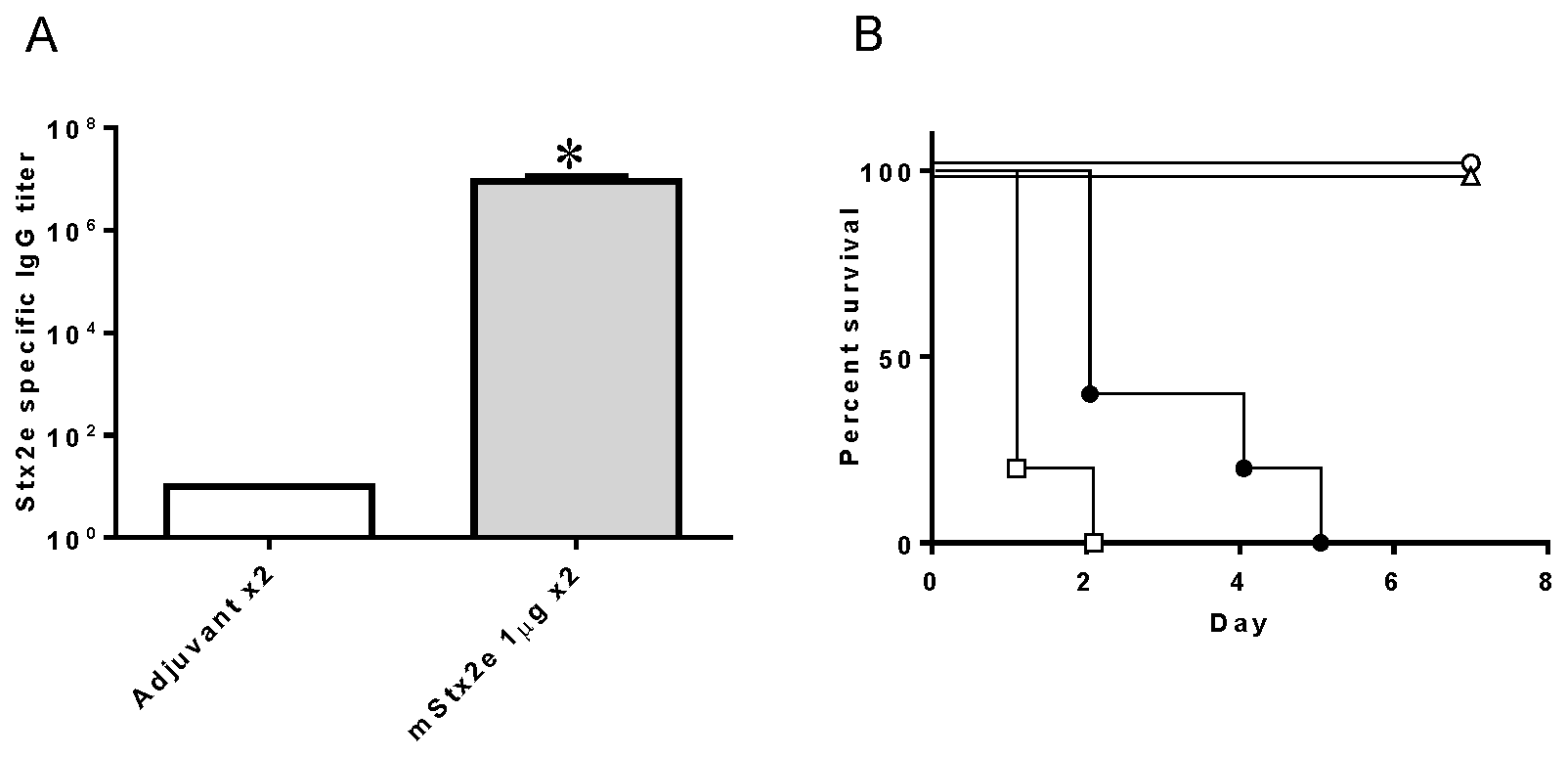
Vaccine effects of attenuated mStx2e in mice. A) Stx2e-specific serum IgG titer in mice immunized with 1 μg of mStx2e (solid column, n=15) and adjuvant only (open column, n=5) determined by ELISA. Data are represented as mean ± SEM values. * Significantly different from adjuvant control group, p < 0.05. B) Survival rate of vaccinated mice against lethal doses of Stx2e challenge. Each mouse was immunized with 1 μg of mStx2e (open symbols, n=15) and intraperitoneally inoculated with 20- (open circle, n=5), 200- (open triangle, n=5), or 2,000-fold (open square, n=5) LD_50_ of Stx2e on day 0. Mice immunized with adjuvant alone (closed circle, n=5) were inoculated with 20-fold LD_50_ of Stx2e.

To evaluate the vaccine effect of mStx2e obtained by these methods, ICR mice were subcutaneously immunized with mStx2e. As shown in Figure 7A, the Stx2e-specific IgG antibody titers in mice immunized with mStx2e were significantly higher than in mice immunized with aluminum hydroxide adjuvant alone (the titers of all 5 mice were less than 10). In the challenge experiment shown in Figure 7B, although all of the mice challenged with 2,000 LD_50_ of Stx2e died, the remaining mice in the mStx2e-immunization group challenged with 200 and 20 LD_50_ of Stx2e survived for at least 1 week with no symptoms. All of the mice immunized with adjuvant alone succumbed to a challenge with 20 LD_50_ of Stx2e within 5 days.

## Discussion

It is well known that some bacterial toxins specifically bind to glycoproteins/glycolipids. Based on these features, affinity purification methods have been developed for some bacterial toxins, such as cholera toxin (CT) and heat-labile enterotoxin (LT) [27], as well as for botulinum type A and B progenitor toxins [28,29]. Affinity purification methods have been established for Stx1 using Gb3 or ovomucoid antigen-immobilized resin [19-21], and commercial Gb3-immobilized resin is available (Globotriose gel, IsoSep AB). Previously, we established an overexpression method for prototype Stx2. Although we confirmed the overexpression of Stx2 in transformants cultured in the presence of lincomycin, we could not purify Stx2 using Gb3-immobilized resin (data not shown). Therefore, to facilitate purification, we prepared recombinant Stx2 by fusing the histidine tag to the C-terminal end of the B subunit (Stx2-His) [24]. Based on these results, we first attempted to prepare histidine-tagged Stx2e (Stx2e-His), since the conventional methods of Stx2e purification are also considerably cumbersome due to the need for a large volume of culture and several chromatography steps [16-18]. However, we failed to express Stx2e-His using our expression method (data not shown). In addition, since Stx2e preferentially binds to Gb4 rather than to Gb3 [10,11], we had to develop an effective method for purifying non-tagged Stx2e using a different approach from that used to purify other Shiga toxins.

We chose three commercially available resins with an immobilized sugar component of Gb4. D-galactose gel is used for the purification of LT and CT [27], whereas lactose gel is available for the purification of botulinum type A and B hemagglutinin-positive progenitor toxins [28,29]. Furthermore, N-acetylgalactosamine gel was chosen because GalNAc is the terminal sugar of Gb4. Among these resins, only D-galactose gel specifically bound Stx2e. 

Bound Stx2e was eluted from the resin by PBS containing 4.5 M MgCl_2_, but was not eluted by PBS containing galactose or lactose or by glycine-HCl buffer. Although MgCl_2_ is a much less expensive reagent than the others, the disadvantage of using MgCl_2_ is that in high concentrations it causes the sample volume to increase during dialysis (in the case of 4.5 M, an approximately 2.5-fold increase in the original eluent volume is observed), leading to dilution of the protein in the eluent. However, the concentrations of the Stx2e eluents that were pooled from the first three fractions (total of 6 ml per run) were sufficiently high after dialysis (14 ml at 0.85–2.8 mg/ml), indicating that our expression method can overcome this disadvantage. 

Although Stx2e bound to α-galactose gel which was also immobilized α-galactose with resin, the amount of Stx2e binding to α-galactose gel was considerably lower than the binding to D-galactose gel. In addition, the fact that bound Stx2e was not eluted from D-galactose gel by buffer containing galactose indicates that Stx2e recognizes other structures in the D-galactose gel, although the structures of other crosslinking components in α-galactose gel have not been disclosed by the manufacturer, except that there is no divinyl sulfone. According to the manufacturer’s instruction for Immobilized D-Galactose Gel, this resin is crosslinked at the first carbon of the α-D-galactose with a divinyl sulfone group. β-mercaptoethanol coupled to divinyl sulfone-activated agarose is designated as TAR and is used in thiophilic interaction chromatography to purify immunoglobulins [26,30]. Immunoglobulins are adsorbed to TAR under a high concentration (0.5 M) of one of the following sulfates; (NH_4_)_2_SO_4_, Na_2_SO_4_, K_2_SO_4_ or MgSO_4_ and are then eluted by decreasing the concentrations of these salts. We confirmed that Stx2e bound to TAR as well as to D-galactose gel. In this study, we used PBS containing 0.5 M NaCl to avoid nonspecific adsorption of contaminants against resin, not to bind Stx2e hydrophobically. Although the thiophilic interaction is distinguished from hydrophobic interactions by the observation that the former reduces γ-globulin binding by increasing the concentration of NaCl [30], we confirmed that Stx2e bound to both TAR and D-galactose gel using buffer with or without NaCl (data not shown), indicating that Stx2e bound to the divinyl sulfone group via a different binding mechanism from that employed in thiophilic interaction chromatography. However, the fact that TAR-purified Stx2e contained some minor contamination, which was not found in Stx2e purified by D-galactose gel, suggests that coupled α-galactose may help improve the binding specificity of Stx2e. In addition to the high binding capacity of Stx2e, D-galactose gel and TAR also have the important advantage of precluding the need to consider any protease contaminants as in the case with glycoprotein-based ligands.

The prototype Stx2 did not bind to TAR as shown in Figure 5 and 6. Furthermore, using mutant Shiga toxins, we found that Stx2e interacted with divinyl sulfone mainly through the asparagine at amino acid 17 in its B subunits. The fact that mutant Stx2 (especially the mutant containing D17N in the B subunits) possessed binding ability to TAR supports our conclusion and indicates the possibility of applying our technique to the simple purification of mStx2 (if the D17N mutation is introduced into the B subunits). According to the report by Tyrrell et al, Stx2e whose B subunits had double mutations (Q64E+K66Q) caused a selective loss of Gb4 binding, but that whose B subunits had the N17D mutation did not [31]. In our experiments, the Q64E+K66Q mutant did not reduce the binding to TAR (data not shown), indicating that the Stx2e binding to divinyl sulfone, which is associated with the asparagine 17, is not the same binding profile with that to Gb4.

Some types of attenuated Stx2e are good candidates for vaccine antigens against edema disease in piglets [12-15]. Since edema disease occurs in weaned piglets, which are immunologically immature, maternal immunization represents another good strategy for conferring maternal antibodies to piglets. Recently, Oanh et al. showed that piglets born from sows vaccinated with Stx2e toxoid during the pregnant period had Stx2e-specific maternal antibodies in their sera, even at 1 month post-weaning, and were protected from Stx2e challenge [15]. However, to enable the practical use of Stx2e toxoid via maternal immunization, cost-effective, large-scale preparation of this toxoid is essential. The mStx2e obtained in the present study conferred sufficient vaccine effects on mice, indicating that our expression and purification methods for Stx2e will be beneficial for large-scale preparation of Stx2e toxoid vaccine in a simple, cost-effective manner.

## Supporting Information

Table S1 **Oligonucleotide primers used in this study.**
(XLS)
